# Aqueous Extract of Brazilian Berry (*Myrciaria jaboticaba*) Peel Improves Inflammatory Parameters and Modulates *Lactobacillus* and *Bifidobacterium* in Rats with Induced-Colitis

**DOI:** 10.3390/nu11112776

**Published:** 2019-11-15

**Authors:** Juliana Kelly da Silva-Maia, Ângela Giovana Batista, Cinthia Baú Betim Cazarin, Edilene Siqueira Soares, Stanislau Bogusz Junior, Raquel Franco Leal, Maria Alice da Cruz-Höfling, Mário Roberto Maróstica Junior

**Affiliations:** 1 Department of Nutrition, Center for Health Sciences, Federal University of Rio Grande do Norte, Rio Grande do Norte, Rua das Artes—Lagoa Nova, Natal, RN 59075-000, Brazil; 2 Department of Food and Nutrition, School of Food Engineering, University of Campinas, Rua Monteiro Lobato, Campinas, SP 13083-862, Brazil; 3 Department of Food and Nutrition, Federal University of Santa Maria—UFSM—Campus Palmeira das Missões, Av. Independência, nº 3751, Palmeira das Missões, RS CEP 98300-000, Brazil; 4 Department of Biochemistry and Tissue Biology, Institute of Biology, University of Campinas, Campinas, SP 13083-862, Brazil; 5 São Carlos Institute of Chemistry (IQSC), University of São Paulo (USP), São Carlos, SP 13566-590, Brazil; 6 School of Medical Sciences, University of Campinas, Campinas, SP 13083-862, Brazil

**Keywords:** antioxidant, jaboticaba, gut inflammation, microbiota, infusion

## Abstract

Natural compounds could be a complementary alternative to inflammatory bowel disease (IBD) management. This study determined the effects of an aqueous extract of *Myrciaria jaboticaba* peel (EJP) (50 g L^−1^) on 2,4,6-trinitrobenzenesulfonic acid-induced colitis. Wistar rats were randomized into five groups: HC—healthy control, CC—colitis control, DC—drug control, SJ—short-term treatment with EJP, and LJ—long-term treatment with EJP. The EJP treatments reduced body weight loss, stool consistency score, and spleen enlargement. Gut microbiota was modulated through increased *Lactobacillus* and *Bifidobacterium* counts after EJP treatment. Short-chain fatty acids were also higher in the EJP treatment groups. The antioxidant enzyme activities were greater than CC or DC controls. Myeloperoxidase activity (LJ), inducible nitric oxide synthase (LJ/SJ), and intercellular adhesion molecule (SJ) levels were lower than in the CC group. EJP decreased histological scoring, mucosal thickness, and preserved the crypts and histological structure. Therefore, EJP showed beneficial effects and could be potentially used as an adjuvant in IBD treatment.

## 1. Introduction

Inflammatory bowel diseases (IBD) are debilitating, relapsing, and chronic pathologies associated with disruption of intestinal epithelial barrier function and mucosal inflammation [1]. The etiology of IBD is not fully understood, but interactions between genetic and environmental factors, gut intestinal microbiota, and genetic and defective immune response have been mentioned [2]. In addition to epithelial barrier disruption, increased oxidative stress caused by an increase in pro-oxidant molecules has been identified as an important mechanism in the induction and progression of ulcerative colitis (UC) [1,3].

Therapies are focused on reducing inflammation and restoring intestinal barrier function. Moreover, studies have confirmed the effectiveness of natural compounds in counteracting IBD [1]. Evidence suggests that their protective and therapeutic effects are on account of their anti-inflammatory and immunoregulatory actions, antioxidative stress, and modulation of intracellular signaling transduction pathways as well as their ability to improve gut microbiota [1,4].

Jaboticaba (*Myrciaria jaboticaba* (Vell.) O. Berg) is a native Brazilian berry, whose dark-purple peels are rich in phenolic compounds, mainly anthocyanins. Previous studies using in vitro and in vivo models have shown that jaboticaba peels present antioxidant and anti-inflammatory properties [5,6]. In addition, literature has shown that anthocyanins have both protective and therapeutic functions over IBD [7].

The purpose of the present study was to investigate the potential of an aqueous infusion of jaboticaba peels to counteract the effects of trinitrobenzenesulfonic acid-induced colitis in rats. In this context, this is the first study that investigates the effects and mechanism of action promoted by the intake of jaboticaba peel aqueous extracts and their use as potential adjuvants to treat intestinal inflammation caused by trinitrobenzenesulfonic acid-induced colitis in rats, mimicking IBD.

## 2. Materials and Methods

### 2.1. Aqueous Extract of Jaboticaba Peel (EJP)

*Myrciaria jaboticaba* peel powder (JPP) was obtained by oven-drying (96 h with air circulation at 40 °C) and milling (Marconi, model MA 630/1, Piracicaba, SP, Brazil) [8]. To prepare the aqueous extract, JPP was immersed in boiling water at 100 °C (212 °F) at 50 g L^−1^ concentration and allowed to rest for 30 min at room temperature (22 ± 2 °C), with eventual mixing after 15 min. The resulting infusion was filtered through a paper filter (18 mm) coupled to a vacuum system [8]. In our previous study that characterized the extract, EJP demonstrated antioxidant capacity according to Ferric Reducing Antioxidant Power (FRAP) and Oxygen Radical Absorbance Capacity (ORAC) assays (353.6 ± 14.8 and 153.8 ± 14.9 mmol Trolox equivalent g^−1^ peel, respectively). In addition, cyanidin-3-*O*-glucoside, delphinidin-3-*O*-glucoside, gallic acid, rutin, myricetin, and quercetin were identified as the main polyphenols in this extract. [8].

### 2.2. Study Design

Male Wistar rats (*Rattus norvegicus*, 21-day-old) acquired from the Multidisciplinary Center of Biological Investigation (CEMIB/UNICAMP) were housed in individual cages under standardized conditions in an animal facility and had unrestricted access to commercial chow (Presence^®^, Evialis do Brasil Nutrição Animal, Ceará, Brazil) as well as tap water. According to the manufacturer, the proximate composition of 100 g of chow was: 23 g protein, 4.5 g lipids, 63.5 g carbohydrates, and 5 g dietary fiber. The list of ingredients of the commercial chow included soybean meal, ground whole corn, extruded whole soybean, wheat bran, wheat flour, corn gluten meal, rice bran, meat and bone meal, fish meal, calcitic limestone, sodium chloride, iron sulfate, copper sulfate, manganese monoxide, zinc oxide, calcium iodate, cobalt sulfate, sodium selenite, vitamin A, vitamin D3, vitamin E, vitamin K, vitamin B1, vitamin B2, niacin, pantothenic acid, vitamin B6, folic acid, biotin, vitamin B12, choline chloride, lysine, methionine, threonine, propionic acid, silicon dioxide, and urea-formaldehyde.

The experiment was approved by the Ethics Committee for the Use of Animals in experimentation (CEUA-UNICAMP #3199-1, Campinas, SP, Brazil) and followed the National Institutes of Health guide for the care and use of laboratory animals (NIH Publications No. 8023).

After one week of acclimatization, the animals were randomized into five groups (*n* = 8/group, except group 3, *n* = 6). Group 1 (HC) comprised of healthy intact animals; group 2 (CC), untreated animals with colitis; group 3 (DC) was treated with mesalazine drug solution (100 mg kg^−1^ dissolved in distilled water) from the sixth and seventh experimental weeks [9]; group 4 (SJ) received short-term EJP intake during the sixth and seventh experimental weeks, and group 5 (LJ) received long-term EJP drinking from the second experimental week to the seventh experimental week (Supplementary Materials, Figures S1 and S2). The EJP and mesalazine solutions were offered instead of water to the animals. Every two days, EJP was freshly prepared and replaced, rats were weighed and the food/liquid intake was controlled. Colitis was induced by a single intracolonic injection of 0.2 mL of 5% 2,4,6-trinitrobenzenesulfonic acid (TNBS) in 50% ethanol at the beginning of the seventh experimental week in groups 2, 3, 4, and 5 following validated protocol [10]. After colitis induction, stool consistency was scored during the first week. Score 1 was assigned for stool that showed pale color, visible mucus, softness, and fecal material adherent to the perianal area; score 2 for stool that did not have normal texture or pellet shape (unformed); score 3 for liquid stool; and 4 was assigned for stool with visible blood. At the end, the total score was summed for each animal [11]. The animals were euthanized by exsanguination via cardiac puncture after being anesthetized with ketamine chloride (Anasedan^®^) and xylazine chloride (Dopalen^®^) (Fortvale, Valinhos, SP, Brazil) (40 and 5 mg kg^−1^, respectively) at the end of the seventh experimental week.

#### 2.2.1. Colon Macroscopic Evaluation

Colon macroscopic evaluation—Ulceration index—Colon was removed and kept in an iced bath. Later, the tissue was longitudinally opened and cleaned with 0.9% saline solution and weighed (mg). The lengths of the colon as well as the colon ulceration region were measured (cm). In relation to the ulceration indexes, the colon was scored as described by Sanchez-Hidalgo et al. [12]. The spleen was also weighed and the result normalized by animal weight.

#### 2.2.2. Cecal Content Analyses

Microbiota analysis—The cecal content (*n* = 6/group) was quickly removed, kept in an iced recipient (bath). The analysis was done as described in a previous work [13]. In short, the cecal content was properly diluted in peptone water and spread on plates with de Man, Rogosa and Sharpe (MRS) agar media appropriate for the growth of *Lactobacillus*, bifidobacteria, enterobacteria, and total aerobic bacteria. The plates were then incubated for 24–48 h. The colony-forming units (CFU) per gram of cecal content were calculated and the results expressed as log^−10^ values of the CFU g^−1^ [13].

Short-chain fatty acids (SCFA)—This analysis in the cecal content was performed as described in our previous study and results were expressed as nmol butyric/acetic/propionic acid g^−1^ feces [13].

#### 2.2.3. Colon Analysis

Histopathological assessment—The samples of colon tissue (distal colon, 7–8 cm from anus, 0.5 × 0.5 cm) were selected and fixed in 10% formalin and then routinely processed for paraffin embedding (Paraplast^®^, Sigma Aldrich, St. Louis, MO, USA). Sections (5 µm thickness) were mounted with Entellan in histological slides (*n* = 6–8 rats/group, 1 section/animal, total of 6–8 sections per group). After dewaxing, the slides were processed for staining with hematoxylin-eosin (H&E). A blind evaluation of the tissue damage was made by trained professionals following the criteria described by Krause et al. [14]. Thickening of the colon tissue was evaluated through measures of mucosa plus submucosa and muscularis mucosae (15 different fields per slide in each animal, 90–120 fields per group) using the ImageJ 1.38 software (US National Institutes of Health).

The remaining segment of the colon was cut into small pieces and frozen in liquid nitrogen for subsequent analyses. The tissue was used to prepare colon homogenate according to the analysis protocol.

#### 2.2.4. Lipid Peroxidation and Antioxidant Enzyme Activities in the Colon Tissue

Thiobarbituric acid reactive substances (TBARS)—After cleaning, colon segment was cut in small pieces and frozen in liquid nitrogen to avoid peroxidation (n = 6–8/group). Lipid peroxidation assessment was done according to the Ohkawa et al. [15] method.

Antioxidant enzyme activity—The colon homogenate in phosphate-buffered saline (PBS), pH 7.4, was prepared and the supernatant was used to perform antioxidant enzyme activity assays after protein concentration measurement [16]. The following antioxidant enzyme assays were carried out as described in detail in previous studies [17]: Superoxide dismutase activity (SOD) [18], glutathione peroxidase activity (GPx) [19], glutathione reductase activity (GR) [20], and catalase (CAT) [21] assays.

#### 2.2.5. Inflammation Markers

Evaluation of neutrophil accumulation by myeloperoxidase activity (MPO)—The colon homogenates of all experimental groups (n = 6–8/group) were prepared with PBS and hexadecyltrimethylammonium bromide (HTAB, Sigma-Aldrich), centrifuged (10,000× *g*, 15 min, at 4 °C) and the supernatant was used to evaluate MPO activity according to Krawisz et al. [22] with modifications in volumes in a microplate assay.

Western blotting—The western blotting analysis was performed for the evaluation of protein expression of inducible nitric oxide synthase (iNOS) and intercellular adhesion molecule 1 (ICAM-1), both from Santa Cruz Biotechnology, Santa Cruz, CA, USA. For quantification, the pixels’ density of each band was determined using the ImageJ 1.38 software (US National Institutes of Health). Endogenous control (β-actin, Sigma-Aldrich, St. Louis, MO, United States) was used to normalize the results.

ELISA assay (IL-1β, IL-6, IL-10, tumor-necrotic factor-α (TNF-α), and monocyte chemoattractant protein-1 (MCP-1))—A multiplex ELISA kit (Merck Millipore, Burlington, MA, USA) was used to evaluate the levels of the interleukins, IL-1β, IL-6, IL-10, TNF-α, and MCP-1, in the colon homogenates of the animals. The assay was performed according to the manufacturer’s instructions (*n* = 6–8/group) in colon homogenate.

### 2.3. Statistical Analyses

Data were expressed as the mean value ± standard error of means (SEM). The GraphPad Prism 5.0 (GraphPad Software, Inc., La Jolla, CA, USA) software was used to analyze the data. The significance of the data was determined using one-way ANOVA and Tukey’s post hoc test at *p* < 0.05, except for the analysis of liquid intake, body weight, and stool score, in which two-way ANOVA followed by Bonferroni post hoc test was used.

## 3. Results

The amount of liquid ingestion was not strongly affected by colitis induction. Seven days after TNBS treatment, all groups showed the same liquid intake level, except the LJ group which had the highest liquid intake level (Table 1). However, only the extract-treated groups were given (poly)phenolic compounds daily: 141.1 ± 16.0 and 151.4 ± 20.0 mg gallic acid equivalent (GAE) kg^−1^ for SJ group before and after colitis induction, respectively, and 215.1 ± 31.7 and 208.0 ± 9.7 mg GAE kg^−1^ for LJ group before and after colitis induction, respectively. In humans, an equivalent dose would be on average 11 to 17 mg GAE kg^−1^ [23].

**Figure 1:**
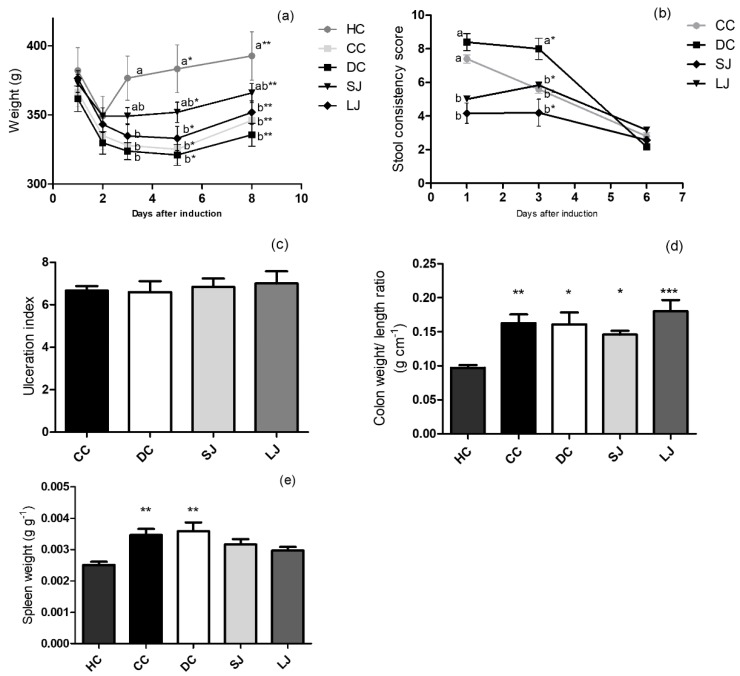
Induced colitis macroscopic markers. (**a**) Body weight (g); (**b**) stool consistency score; (**c**) colon ulceration index; (**d**) colon weight/ length ratio (g cm^−1^); (**e**) spleen/ body weight ratio (g g^−1^). HC = healthy control; CC = colitis control, untreated; DC = drug (mesalazine) control; SJ = short-term EJP extract treatment; and LJ = long-term EJP extract treatment. Aqueous extract of Jaboticaba peel (EJP). Results are expressed as the mean ± SEM (*n* = 6–8). Different letters/symbols indicate significant difference (*p* ≤ 0.05) according to two-way ANOVA and Bonferroni test in Figures (a) and (b). * Indicates significant difference relative to HC group by one-way ANOVA and Tukey’s test in Figures (c)–(e) (1 code = *p* < 0.05; 2 codes = *p* < 0.01; and 3 = *p* < 0.001).

Due to the 12 h-fasting prior TNBS rectal instillation, all groups showed a decline in body weight in the first day after induction and thus body weight loss was similar among the illness groups (CC, DC, SJ, and LJ). However, the SJ and LJ groups showed a trend to minimize the weight loss, especially the SJ group (Figure 1a). The stool consistency score was found to be directly related to the severity of colon injury; EJP-treated groups (SJ and LJ) showed lower stool consistency scores than the CC and DC groups one day after colitis induction; that difference was maintained until the third day relative to the DC group (Figure 1b). On the other hand, colon ulceration index as well as the ratio of colon weight/length were similar among colitis-induced groups, irrespective of treatment (Figure 1c,d). Spleen weight was significantly higher in the CC and DC groups compared with the health control group (HC). In contrast, SJ and LJ showed no statistical difference from HC (*p* = 0.071 and 0.331, respectively), suggesting that EJP infusion attenuates splenomegaly (Figure 1e).

**Figure 2:**
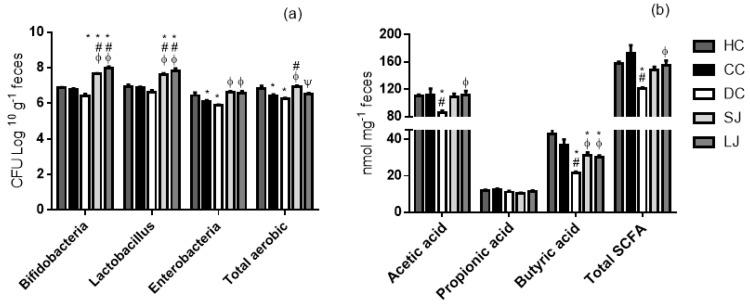
Microbiota and short-chain fatty acid (SCFA) production in cecal content. (**a**) Cecal microbiota (CFU log 10 g^−1^ feces) and (**b**) SCFA (nmol mg^−1^ feces). HC = healthy control; CC = colitis control; DC = drug (mesalazine) control; SJ = short-term EJP extract treatment; and LJ = long-term EJP extract treatment. Aqueous extract of Jaboticaba peel (EJP). Results are expressed as the mean ± standard error (*n* = 6–8). * Indicates significant difference from HC group, # from CC group, Φ from DC, and Ψ from SJ by one-way ANOVA and Tukey’s test (1 code = *p* < 0.05; 2 codes = *p* < 0.01; and 3 codes = *p* < 0.001).

The EJP intake for short- or long-term (SL or LJ) treatment stimulated bifidobacteria and *Lactobacillus* growth (Figure 2a). Likewise, SJ and LJ intakes also kept the enterobacterial population similar to that of the healthy animals (HC). The counts of enterobacteria were significantly increased when compared to the drug-treated animals (DC) in EJP-treated groups. The SJ treatment was able to boost the total aerobic growth, which was higher than DC and CC. However, LJ did not impair aerobic microbiota, since both the treatments (SJ and LJ) showed a statistically equal count of total aerobics compared to healthy rats (HC), while CC and DC were lower than HC.

The SCFA production, resulting from microbiota metabolism, was overall reduced in the mesalazine-treated group (DC). The SJ and LJ groups had greater butyric acid levels compared with DC. In addition, LJ treatment showed the same effect on acetic acid levels and total SCFA that was found significantly higher than in the animals treated with mesalazine (DC). There was no difference for propionic acid among the groups (Figure 2b).

**Figure 3:**
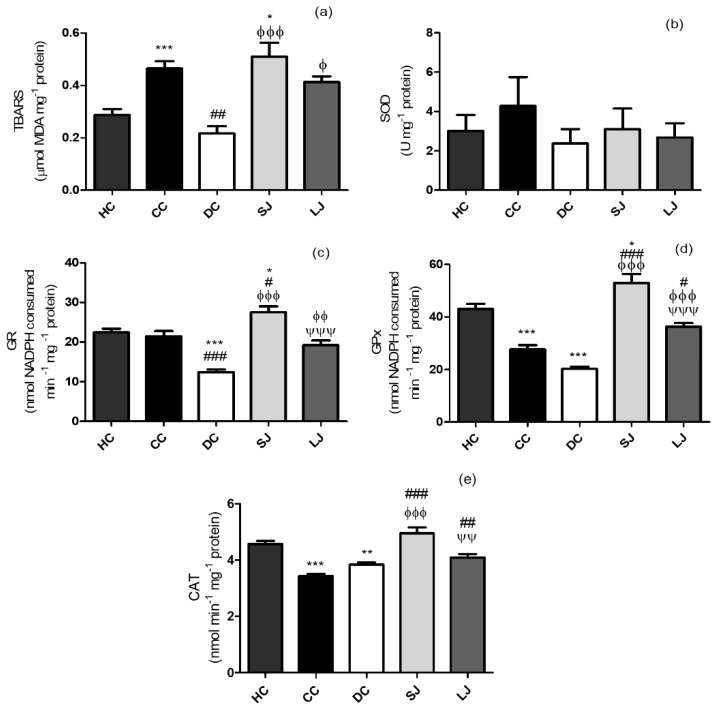
Lipid peroxidation and antioxidant defenses of colon. (**a**) Thiobarbituric acid reactive substances assay - TBARS (µmol malondialdehyde-MDA); (**b**) SOD (U); (**c**) GR (nmol nicotinamide adenine dinucleotide phosphate - NADPH consumed min^−1^); (**d**) GPx (nmol NADPH consumed min^−1^) and (**e**) CAT (nmol min^−1^) mg^−1^ protein. HC = healthy control; CC = colitis control; DC = drug (mesalazine) control; SJ = short-term EJP extract treatment; and LJ = long-term EJP extract treatment. Aqueous extract of Jaboticaba peel (EJP). Results are expressed as the mean ± standard error (*n* = 6–8). * Indicates significant difference from HC group, # from CC group, Φ from DC, and Ψ from SJ by one-way ANOVA and Tukey’s test (1 code = *p* < 0.05; 2 codes = *p* < 0.01; and 3 = *p* < 0.001).

The TBARS measurement is indicative of lipid peroxidation, and oxidative stress is critical among the causes involved in IBD [15]. TBARS values in the LJ group were similar to HC and CC, and SJ group did not differ from the CC group (Figure 3a).

Our results indicated a distinct time-dependent impact of treatment with EJP on SOD, GR, GPx, and CAT. There was no alteration in SOD activity in the colon among the groups (Figure 3b). Short-term protocol stimulated GR and GPx activities, being significantly different from CC (GR and GPx) and from DC (GR) (Figure 3c,d). The treatments with EJP also increased CAT activity, whereas SJ and LJ treatments showed higher CAT activity than CC. SJ also showed higher CAT activity than the DC group (Figure 3e).

**Figure 4:**
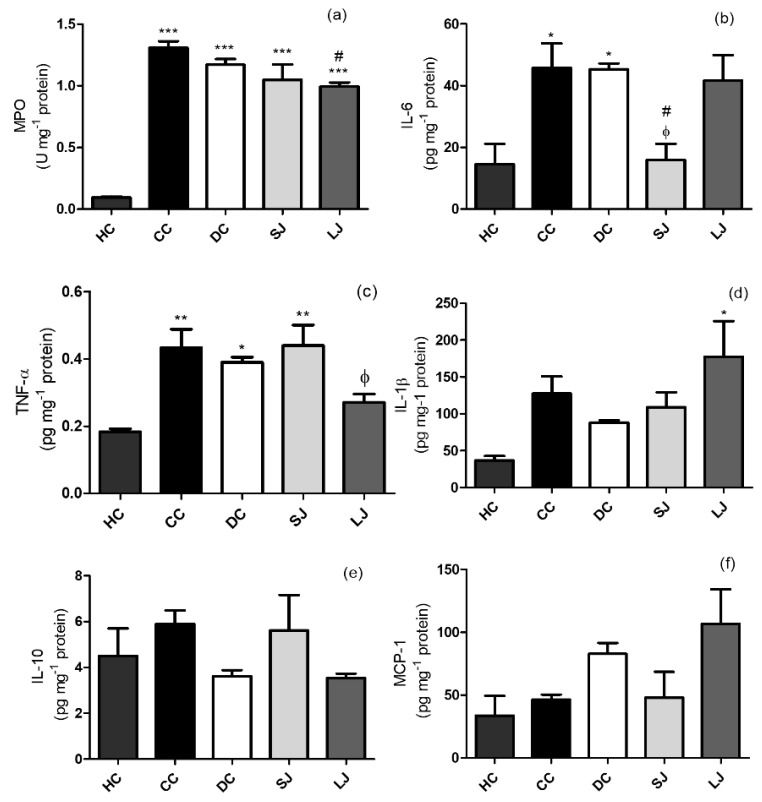
Inflammation markers. (**a**) Myeloperoxidase (MPO) activity (U) mg^−1^; (**b**) IL-6; (**c**) tumor-necrotic factor-α (TNF-α); (**d**) IL-1β; (**e**) IL-10; and (**f**) Monocyte chemoattractant protein-1 (MCP-1) pg mg^−1^ protein in the colon. HC = healthy control; CC = colitis control; DC = drug (mesalazine) control; SJ = short-term EJP extract treatment; and LJ = long-term EJP extract treatment. Aqueous extract of Jaboticaba peel (EJP). Results are expressed as the mean ± standard error (*n* = 3–5). * Indicates significant difference from HC group, # from CC group, and Φ from DC by one-way ANOVA and Tukey’s test (1 code = *p* < 0.05; 2 codes = *p* < 0.01; and 3 codes = *p* < 0.001).

The LJ group showed lower MPO concentration than the CC group (Figure 4a). The ELISA analysis showed that SJ treatment decreased IL-6 to the same level than shown by the healthy group (HC), and significantly lower than CC and DC (Figure 4b). In addition, colonic TNF-α in LJ showed a trend to reduce their levels when compared to CC (*p* = 0.0538); and it was lower than the DC group and similar to the HC group (Figure 4c). No other significant results regarding IL-1β, IL-10, and MCP-1 were found (Figure 4d–f).

**Figure 5:**
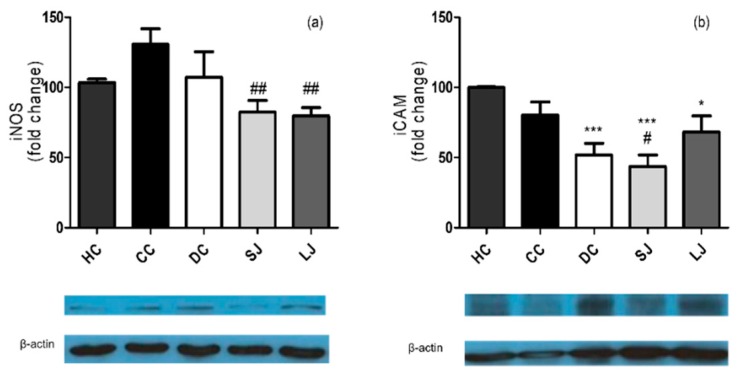
Western blotting inflammation markers in the colon. (**a**) Inducible nitric oxide synthase (iNOS) and (**b**) Intercellular adhesion molecule 1 (ICAM-1) (fold change). HC = healthy control; CC = colitis control; DC = drug (mesalazine) control; SJ = short-term EJP extract treatment; and LJ = long-term EJP extract treatment. Results are expressed as the mean ± error standard (*n* = 3–5). * Indicates significant difference from HC group and # from CC group by one-way ANOVA and Tukey’s test (1 code = *p* < 0.05; 2 codes = *p* < 0.01; and 3 codes = *p* < 0.001).

The results from western blotting confirmed the anti-inflammatory action of EJP in the colon, since the LJ and SJ groups demonstrated lower iNOS expression than the CC group, while only SJ showed lower ICAM expression than the CC group (Figure 5a,b).

**Figure 6:**
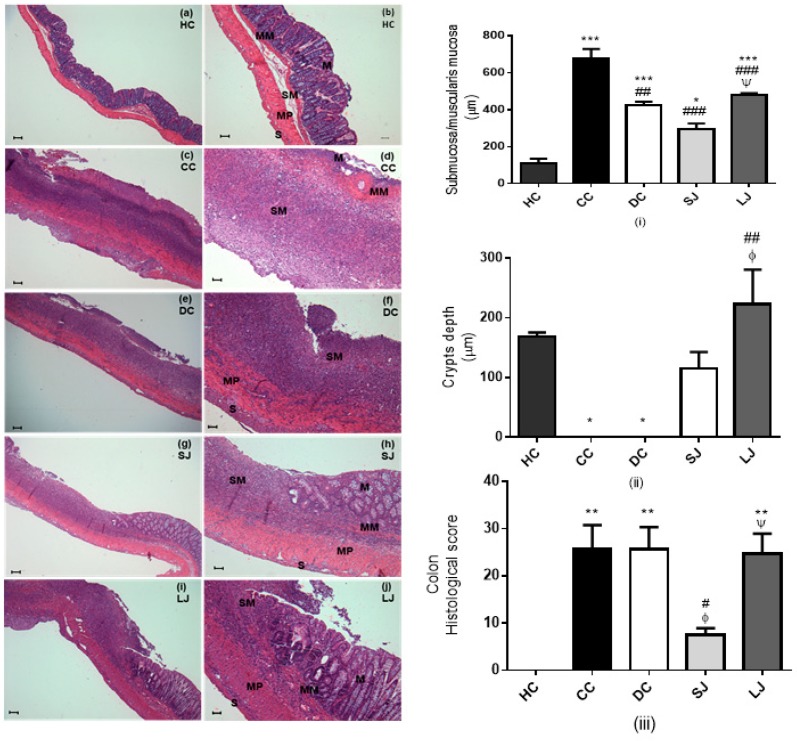
Histopathological assessment. Light micrographs of hematoxylin-eosin (HE)-stained colon from HC = healthy control (**a**,**b**); CC = colitis control (**c**,**d**); DC = drug (mesalazine) control (**e**,**f**); SJ = short-term EJP extract treatment (**g**,**h**); and LJ = long-term EJP extract treatment (**i**,**j**). Scale bars = 100 µm (a, c, e, g, i); 50 µm (b, d, f, h, j); (**i**) submucosa/muscularis mucosa thickness (µm); (**ii**) crypt depth (µm); (**iii**) Histological score. Aqueous extract of Jaboticaba peel (EJP). Results are expressed as the mean ± error standard (*n* = 6–8). * Indicates significant difference from HC group, # from CC group, Φ from DC, and Ψ from SJ by ANOVA and Tukey’s test (1 code = *p* < 0.05; 2 codes = *p* < 0.01; and 3 codes = *p* < 0.001). Mucosa (M), muscularis mucosae (MM), submucosa (SM), muscularis propria (MP), and serosa (S).

Intestinal inflammation impairs intestinal barrier function due to disruption of epithelial tight junctions, sloughing off epithelial cells of gut mucosa causing crypts erosions and ulcerations [24]. Rats which suffered from colitis induction two weeks earlier (CC group) showed ulcerative colon with erosion of mucosae and complete absence of goblet cells; a huge population of lymphoid cells had permeated; in addition, there was hyalinization of muscularis characterized by marked eosinophilia compared to the HC group (Figure 6a–d). A similar outcome is shown by UC-presenting rats treated with mesalazine (DC group) (Figure 6e,f). Rats treated with EJP for six (LJ) or two (SJ) weeks showed shortened portions of colon ulceration, preservation of crypts and glands lined by goblet and absorptive cells, and submucosa/muscularis mucosae and muscular layers thickness more closely similar to healthy (HC) dimension compared to the CC and DC groups (Figure 6g–j) according to metric analysis of captured images (Supplementary Materials—histological pictures of each group, Figures S3–S6). These results highlighted that histological scoring was lower in SJ than all the other colitis groups (CC, DC, and LJ), and without statistic difference from the HC group [14].

## 4. Discussion

Bioactive compounds have been confirmed as effective in IBD management by their protective and therapeutic actions such as anti-inflammatory and antioxidant effects as well as gut microbiota modulation capacity among the involved mechanisms of action [4]. The present study has also shown that an anthocyanin-rich aqueous extract of jaboticaba peel intake ameliorated colitis symptoms such as body weight loss, morphological changes in the colon segment, high inflammation markers, and stool consistency score. Similar results were found by Pereira et al. [25] in rats with colitis treated with blueberries’ anthocyanins. Additionally, we showed other improvements, for example, a reduction of splenomegaly in EJP-treated rats and maintenance of spleen weight at the level of healthy rats. Apart from the production of blood cells, the spleen is also a lymphoid organ involved in the immune system, and the spleen can become enlarged in response to various settings, including IBD [26].

Another benefit promoted by EJP treatment was related to cecal gut microbiota modulation and colon mucosal preservation. The intake of EJP amended goblet cell population that had been destroyed and detached in the course of colitis-induced ulceration as well as stimulated the growth of bifidobacteria and *Lactobacillus*, besides keeping unchanged the enterobacteria and total aerobic populations compared to healthy animals. Non-pathogenic intestinal microbiota is known to be associated with beneficial functions in hosts, which overall play a protective role against gut inflammation and disease progression [27]. (Poly)phenols can modulate the microbiota and consequently, SCFA formation [28]. Corroborating our findings, Dolara et al. [29] have found predominance of *Lactobacillus* and *Bifidobacterium* spp. in feces of rats, which had been supplemented for 16 weeks with proanthocyanidin-rich red wine extract. In addition, EJP was able to promote butyrate (SJ and LJ) and acetate production (LJ) and total SCFA (LJ) when compared to mesalazine treatment. Mesalazine, also called mesalamine (5-ASA) is a 5-aminosalicylate anti-inflammatory drug used to treat IBD, including ulcerative colitis and Crohn’s disease [30].

The association of reactive oxygen species (ROS) with IBD has been already well established. Herein, lipid peroxidation was increased in colitis groups (CC and SJ) according to the TBARS assay. However, mesalazine was able to protect colon tissues against lipid peroxidation (DC group). In the same way, there was no statistic difference between LJ and HC. This result demonstrated that EJP, when administered as a long-term treatment, has a protective role against oxidative damage. In agreement with these results, apple peel polyphenols were shown to be capable of preventing higher lipid peroxidation caused by experimental IBD [31].

Literature suggests that IBD has been associated with an imbalance between increased reactive species and antioxidant defenses [32] and that EJP treatment could be used as a non-invasive approach in IBD-related oxidative stress management. In the present work, both the EJP-treatment protocols boosted antioxidant enzyme activities, where EJP was found to have the most effect on the activity of GR, GPx, and CAT; only SOD activity remained unaffected. Either way, other studies with jaboticaba peel intake have proved antioxidant effects in in vivo and in vitro models of obesity and subclinical inflammation [5,33]. The treatment with blueberry anthocyanin fraction also boosted the activity of antioxidant enzymes of rats with induced colitis [25], and was observed in the present work as well, while the drug-treated group was not able to improve the antioxidant defense.

Mazzon et al. [34] have shown that green tea extract downregulated ICAM-1 expression and MPO activity in rats submitted to experimentally induced colitis and found similar data as this study. The EJP intake in the LJ treatment group decreased 25% TNF-α expression in colon tissues compared to the CC group (*p* = 0.0538). In addition, EJP reduced TNF-α in the LJ group to a level similar to that observed in the HC group, in addition to reduced MPO activity compared to CC group. Moreover, our findings showed lower iNOS compared to the CC group in both the groups treated with EJP. The data agree with the study by Pereira, Pereira, Figueiredo, Freitas, Dinis and Almeida [25] who have also found lower levels of MPO and iNOS after treatment of colitis with a blueberry fraction. Increased MPO and higher iNOS activity were demonstrated after TNBS-induced colitis [35] and they have been related to the chronicity of the inflammation status. The findings, in general, suggest the anti-inflammatory properties of the jaboticaba peel extract.

The histological healing of mucosal inflammation has been associated with a decline of proinflammatory cytokines [36,37]. Therefore, the observed reduction in inflammatory markers such as MPO, IL-6, TNF-α, iNOS, and ICAM can be attributed to the mucosal improvement findings.

Corroborating our study, grape seed extract significantly decreased mucosal thickness in rats with dextran sulfate sodium (DSS)-induced colitis [38], which indicates relief of inflammation by reduction of inflammatory cell infiltrate. In agreement with our results, no significant effect was evident in distal colon related to qualitative histological severity score [38]. Additionally, the EJP treatment protocol used in our study has prevented TNBS-induced damage to the mucosal crypts and glands to a significant extent, and histological score of colon damage was significantly reduced in SJ-treated animals. In general, there was improvement in the EJP-treated animals, but unexpectedly the shorter protocol was more effective according to histological scores, suggesting a time-independent dose response. Other data have suggested the anti-inflammatory action of either SJ or LJ, as evidenced by the reduction of submucosa and muscularis mucosae thickness, preservation of intestinal crypts, and reduction of inflammatory markers: MPO (LJ), IL-6 (SJ), TNF-α (LJ), ICAM (SJ), and iNOS (LJ and SJ).

The current study is the first investigation on the anti-inflammatory action of jaboticaba peel extract against IBD and this mechanism can be further explored in subsequent researches. Therefore, prospective research can explore molecular responses as expression of inflammatory cytokines and 16S genomic analysis of microbiota from different treatment times to conclude if the acute protocol (SJ) with the jaboticaba peel extract is better for the treatment of IBD. Inflammatory bowel diseases are characterized by alternating remission and exacerbation periods and short-term adjuvant treatment with EJP can be used in the acute phase of inflammation. Other approaches could be implemented to investigate the action of EJP plus mesalazine in IBD treatment as a way to combine the positive effects of both and to optimize the treatment.

## 5. Conclusions

The results demonstrated that an aqueous extract of jaboticaba peel used as an infusion and taken as a beverage can reduce the severity of experimental colitis. The benefits were attributed to its anti-inflammatory effects and capacity to inhibit oxidative stress, stimulate beneficial gut bacteria such as *Lactobacillus* and bifidobacteria, and preserve healthy mucosa, mainly by acute treatment. The effect in the spleen size showed by EJP is further evidence of its immunologic attributes in the combat of colitis-induced systemic inflammation. Results suggest that jaboticaba extract has better effects on the growth of *Lactobacillus* and *Bifidobacterium* microbiota and SFCA production than mesalazine, which is widely used in IBD management, and may be confirmed by future 16S genomic analysis of the microbiota. Therefore, prospective studies could explore the potential of EJP in adjuvant therapy for IBD.

## Figures and Tables

**Table 1:** Liquid intake before and after colitis induction.

Liquid Intake		
mL Day^−1^ Animal^−1^		
GROUPS	Experimental Days *	
	49th	56th
HC	45.8 ± 5.4 ^b^	44.3 ± 7.1 ^b^
CC	37.8 ± 3.4 ^c^	37.9 ± 4.5 ^b^
DC	48.9 ± 10.1 ^b^	42.9 ± 7.7 ^b^
SJ	37.4 ± 4.3 ^c^	40.1 ± 5.3 ^b^
LJ	57.1 ± 8.4 ^a^	55.2 ± 2.6 ^a^

* 49th = before colitis induction; 56th = 7 days after colitis induction. HC = healthy control; CC = colitis control untreated; DC = drug (mesalazine) control; SJ = short-term Aqueous extract of Jaboticaba peel (EJP) extract treatment; and LJ = long-term EJP extract treatment. Results are expressed as the mean ± SEM (n = 6–8). Different letters in the columns indicate significant difference (*p* < 0.05).

## Supplementary Materials

The following are available online at https://www.mdpi.com/2072-6643/11/11/2776/s1. Figure S1: Experimental Design—Time experimental, procedures, groups and treatment description., Figure S2: Light micrographs of hematoxylin-eosin (HE)-stained colon from HC = healthy control, S3: Light micrographs of hematoxylin-eosin (HE)-stained colon from CC = colitis control, S4: Light micrographs of hematoxylin-eosin (HE)-stained colon from DC = drug (mesalazine) control, S5: Light micrographs of hematoxylin-eosin (HE)-stained colon from SJ = short-term EJP extract treatment, S6: Light micrographs of hematoxylin-eosin (HE)-stained colon from LJ = long-term EJP extract treatment
