# Supplementary figures and images for: Aqueous Extract of Brazilian Berry (Myrciaria jaboticaba) Peel Improves Inflammatory Parameters and Modulates Lactobacillus and Bifidobacterium in Rats with Induced-Colitis
Source: Nutrients. 2019 Nov 15;11(11):2776. doi: 10.3390/nu11112776 (PMC6893622; doi:10.3390/nu11112776)

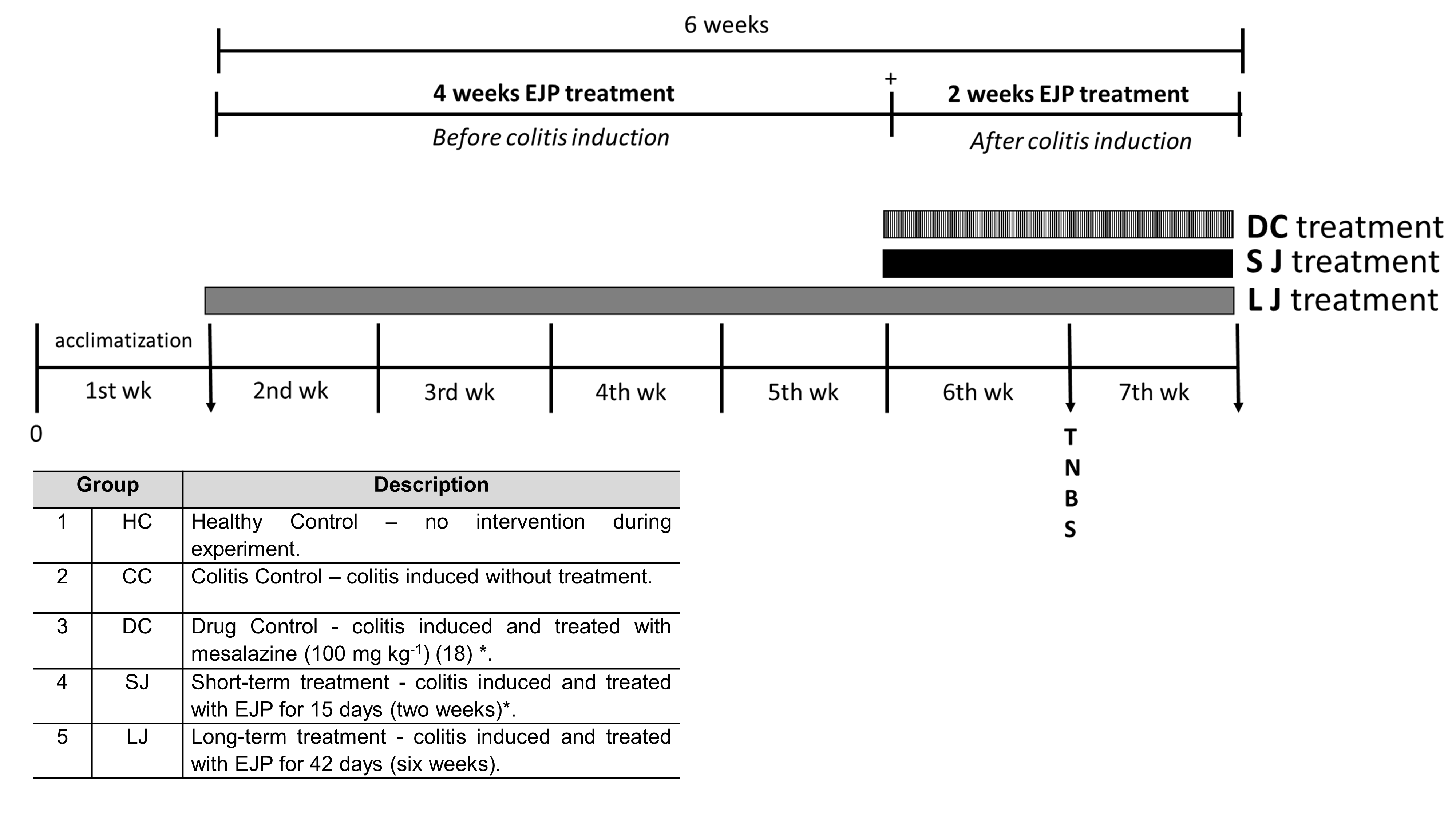

Supplement: Supplementary file 1 [file nutrients-11-02776-s001.zip › Figuras da Figura 6/Fig S1.tif]

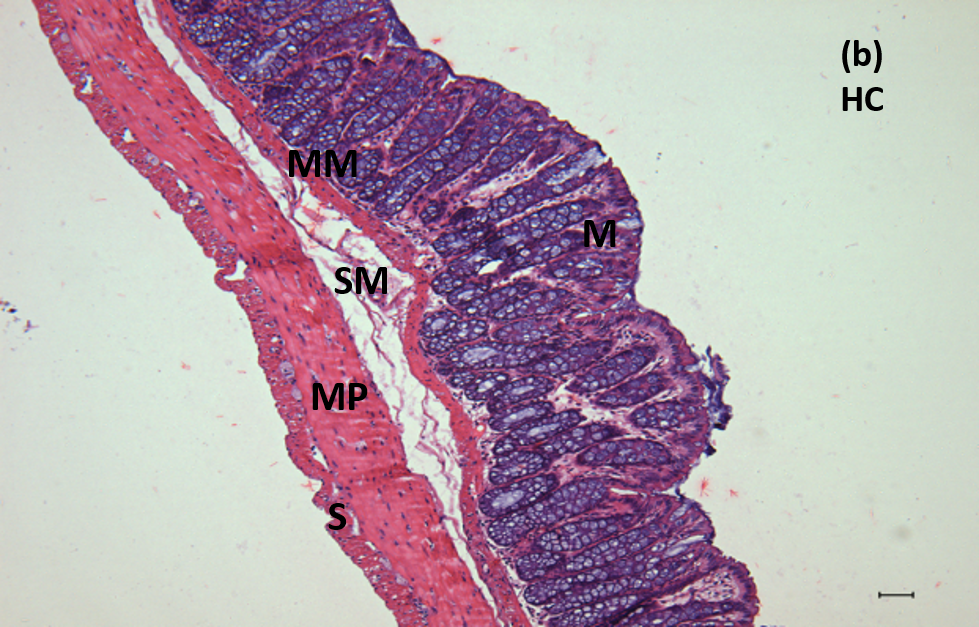

Supplement: Supplementary file 1 [file nutrients-11-02776-s001.zip › Figuras da Figura 6/Fig S2.tif]

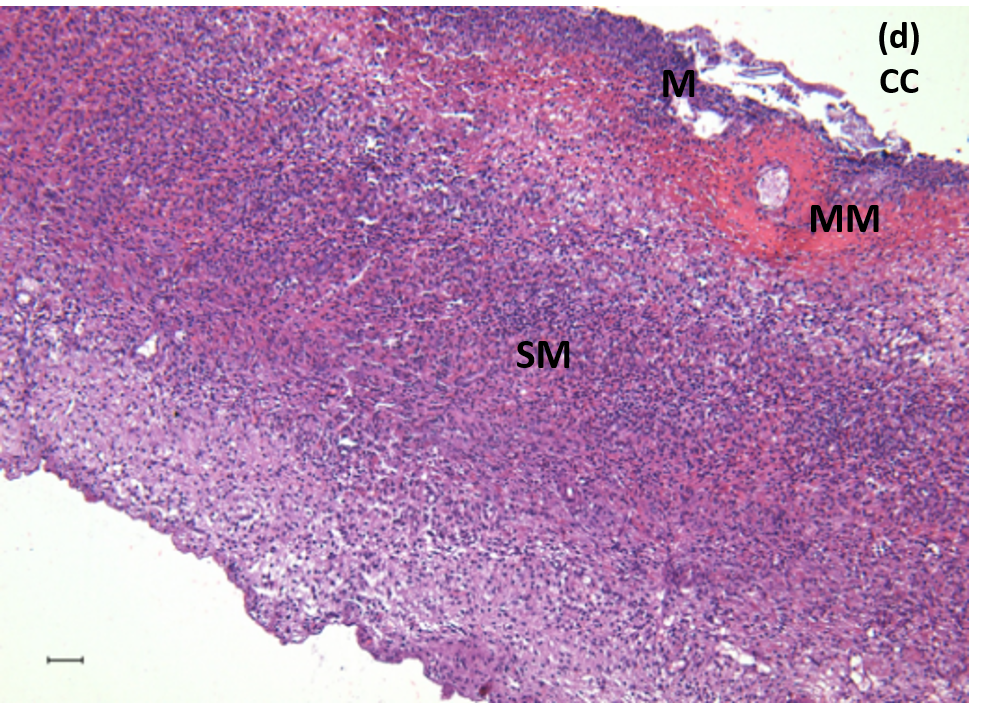

Supplement: Supplementary file 1 [file nutrients-11-02776-s001.zip › Figuras da Figura 6/Fig S3.tif]

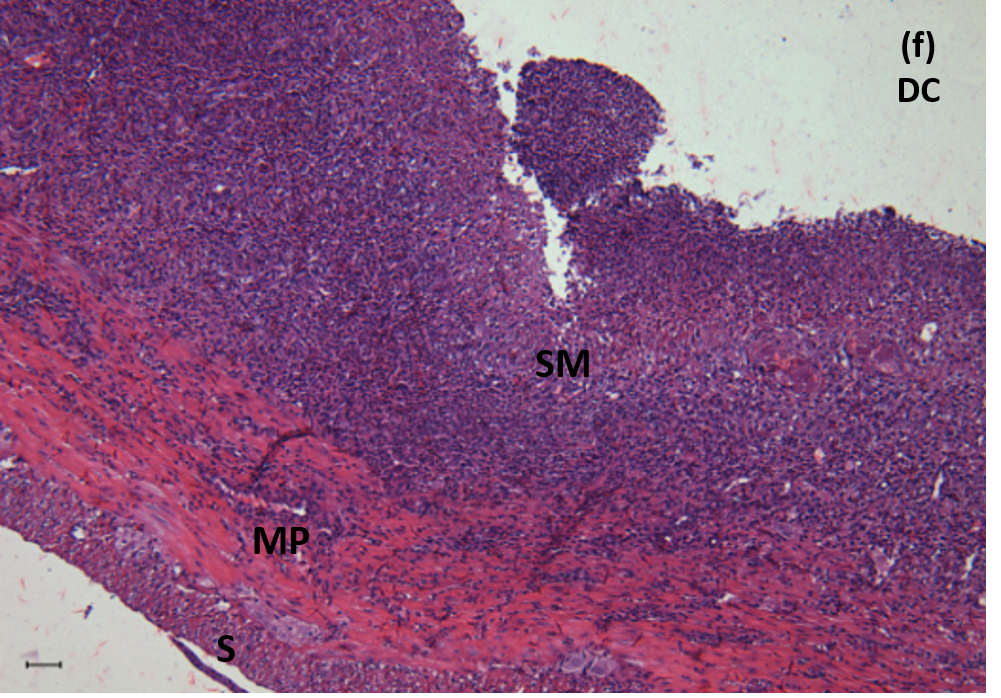

Supplement: Supplementary file 1 [file nutrients-11-02776-s001.zip › Figuras da Figura 6/Fig S4.tif]

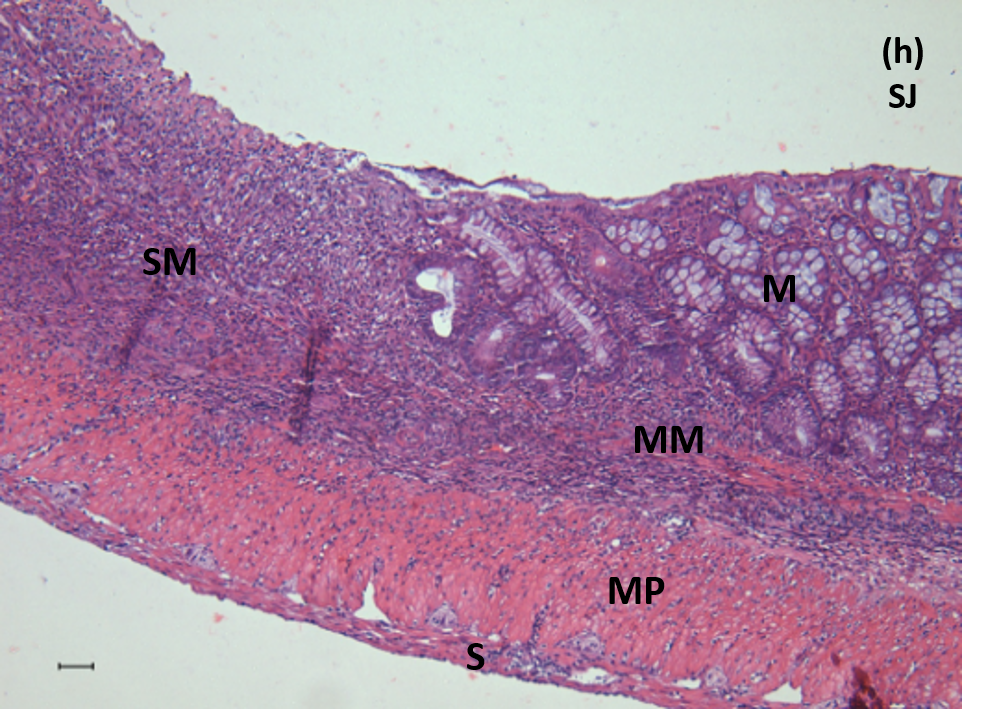

Supplement: Supplementary file 1 [file nutrients-11-02776-s001.zip › Figuras da Figura 6/Fig S5.tif]

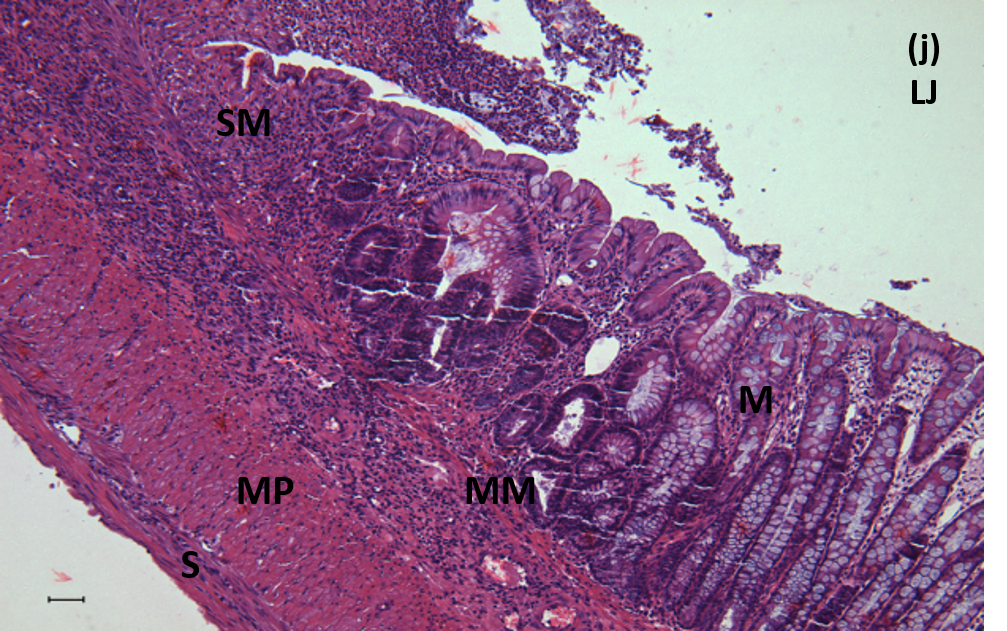

Supplement: Supplementary file 1 [file nutrients-11-02776-s001.zip › Figuras da Figura 6/Fig S6.tif]
